# The impact of the COVID-19 pandemic and associated public health response on people with eating disorder symptomatology: an Australian study

**DOI:** 10.1186/s40337-021-00527-0

**Published:** 2022-01-17

**Authors:** Jane Miskovic-Wheatley, Eyza Koreshe, Marcellinus Kim, Rachel Simeone, Sarah Maguire

**Affiliations:** 1 InsideOut Institute, Central Clinical School, Faculty of Medicine and Health, The University of Sydney, Charles Perkins Centre, Camperdown, NSW 2006 Australia; 2 Sydney Local Health District, St Leonards, NSW Australia

**Keywords:** Eating disorders, Anorexia nervosa, COVID-19, Pandemic, Public health

## Abstract

**Objective:**

People with lived experience of eating disorders (ED) may be particularly vulnerable to the COVID-19 pandemic and associated public health response due to exasperating situations such as social isolation, presence of other mental and physical health conditions, disruptions to treatment, etc. This study investigates the association of the pandemic with ED symptomatology to consider impact and identify risk factors for clinical consideration.

**Methods:**

Participants with self-reported ED diagnosis and/or symptomatology over 16 years were invited to complete an online survey during the first months of the pandemic in Australia. Questions included history of ED, occurrence of co-occurring mental health conditions, change in ED symptoms since the start of the pandemic, and validated measures of ED illness, state mental health and loneliness.

**Results:**

Of 1723 participants (mode age 24.9 years, 91.6% identifying as female, EDE-Q Global Score x = 4.08, SD = 1.18, 79.0% reporting co-occurring mental health condition, predominantly obsessive–compulsive disorder and/or anxiety), 88.0% reported an increase in body image concerns, 74.1% in food restriction, 66.2% binge eating and 46.8% driven exercise during the pandemic. Increased ED symptomatology was associated with poorer state mental health (i.e., depression, anxiety, and stress) and loneliness across the ED symptom profile. Most participants were negatively impacted by various aspects of the public health response, more so for those with more acute ED illness as measured by the Eating Disorder Examination Questionnaire (EDE-Q).

**Conclusions:**

Associated with the COVID-19 pandemic is a mental health crisis, particularly for those with a lived experience of an eating disorder. With 40.5% of participants not having sought formal diagnostic assessment and less than half in treatment, this study provides evidence for the detrimental impact of the pandemic on people with a lived experience of an eating disorder, especially for those not yet supported by the health care system.

**Supplementary Information:**

The online version contains supplementary material available at 10.1186/s40337-021-00527-0.

## Plain English summary

This study investigates the impact of the COVID-19 pandemic and associated public health response on people with a self-reported lived experience of eating disorders across Australia. A nation-wide online survey of 1723 participants aged 16–80 years indicated eating disorders symptoms increased globally including body image concern (for 88% of participants), food restriction (74%) and binge eating (66%), especially for those reporting more acute eating disorder illness, poorer mental health (including depression, anxiety, and stress) and experience of loneliness. Albeit necessary, several pandemic experiences were identified as being particularly associated with more acute eating disorder illness such as changes in daily routine, social media reactions, restricted access to support people, and changes to treatment. As less than half of the participants were in treatment at assessment and over 40% had never sought formal diagnosis or treatment, this study highlights the prevalence of unidentified and unsupported people in the community experiencing increase eating disorder symptoms during this pandemic and the need for clinical awareness in general medical and mental health practice.

**Supplementary Information:**

The online version contains supplementary material available at 10.1186/s40337-021-00527-0.

## Introduction

The coronavirus disease 2019 (COVID-19) was declared a global pandemic in March 2020 by the World Health Organization and as of March 2021 had infected over 117 million people and caused over 2.6 million deaths worldwide [1]. Along with chronic respiratory abnormalities, longer term health implications have already been reported including cardiovascular [2], renal, dermatologic and neurological concerns [3]. Research on the impact of acute respiratory epidemics on vulnerable populations have shown neuropsychiatric linkage between outbreaks and mental disorders [4].

The COVID-19 pandemic has similarities with past outbreaks in terms of global fear due to the virus itself, financial insecurity, and modern quarantine strategies such as mandatory lockdowns and ‘social distancing’ to curtail viral spread. Isolation significantly impacts mental health causing feelings of anxiety, depression, anger, and loneliness, with increased duration in quarantine directly associated with increased symptoms of Post-Traumatic Stress Disorder [5]. Following early warnings from the United Nations about a pandemic-associated mental health crisis [6], population-wide mental health concerns have been reported including a twofold increase in anxiety disorders, a threefold increase in major depressive disorders and suicide risk, and significant increases in binge drinking [7]. While all communities have been impacted socially, financially and psychologically, the pandemic may have significant impact on those most vulnerable, such as people with pre-existing mental health illnesses [8], especially people with lived experience of eating disorders [9].

Eating Disorders (ED) are a range of complex psychological disorders which can lead to significant physical and psychological impairment and are associated with high rates of mortality and low rates of detection and intervention [10–13]. Among the EDs, Anorexia Nervosa has the highest reported mortality rate of any psychological illness [14], with approximately 12 times greater risk of death and 57 times greater risk of suicide as compared to peers [13, 15]. Due to the high level of impairment, chronic health consequence and mortality, people with an ED warrant considered attention during this pandemic.

People with a lived experience of an ED may be particularly vulnerable due to the impact social isolation and loneliness can have on the illness [16]. People with EDs have a complex relationship with food, so issues around food shortages and stock-pilling experienced at the panicked start of lockdowns can lead to worsening symptoms [9]. As compulsive exercise is a common feature and coping strategy in ED’s, closures of health and fitness centres can trigger ED symptoms such as binge-eating, purging, body image concern, excessive exercise and diet pill misuse [17]. Due to the high co-occurrence of contamination fears and obsessive–compulsive symptomatology with EDs [18], fear of disease contamination and increased hygiene measures may also exasperate anxiety and ED symptoms [19]. Research from the early months of the pandemic reported a significant increase in symptoms across all ED diagnoses [20], reactivation of symptoms [21], significant increase in hospital admission for children [22], and interference with the recovery process [23]. As this pandemic moves from initial crisis to more sustained change in our way of life, it is crucial to understand potential consequences of this pervasive experience for those in our community most at risk, especially for those with pre-existing mental and physical health concerns, such as for those with a lived experience of eating disorders.

This study aims to better understand the pandemic experience for individuals experiencing eating disorder symptomatology within the community to identify risk factors and to refine clinical understanding. Primarily, it is hypothesised that the COVID-19 pandemic and associated public health response, while necessary, have had a detrimental impact on the mental health of people with an ED, especially for those with a higher level of illness. Secondarily, it is hypothesised that those with co-occurring mental health conditions, those experiencing higher levels of depression, anxiety and/or stress, those experiencing loneliness, and/or those not actively engaged in treatment during the pandemic will be more negatively impacted, reporting worsening ED symptoms. This was an online community survey to investigate the impact the COVID-19 pandemic has had on people with a lived experience of an eating disorder.

## Method

### Design and setting

This was a national cross-sectional survey of people 16 years and over with diagnosis of an eating disorder (ED) or experiences of ED symptomatology in Australia. A survey design, delivered by a secure online platform REDCap (Research Electronic Data Capture) [24] was chosen due to reach, cost-effectiveness and as a safe mode of delivery during public health orders, with recruitment open July to October 2020 during the second significant wave of cases and widespread public health lockdowns. The online survey was promoted within mental and general health sectors, via social media advertising and national media coverage. The estimated time to complete the survey was 20 min and was only offered in the English language. All participants who completed were entered into a draw to win one of two shopping vouchers worth AUD100 (< 0.001 probability) as a retention strategy. Collected data was deidentified and stored in a secure network data management system compliant with University Research Data Management Policy.

This study was approved by the Human Ethics Review Committee (Royal Prince Alfred Hospital Zone) of the Sydney Local Health District, Australia, protocol number X20-0181. This study did not receive external funding, rather it was conducted by the research team from the InsideOut Institute, University of Sydney, due to the unprecedented opportunity and recognised need.

### Participants

Anyone with a self-reported current or previous clinical diagnosis of an eating disorder [25] or self-reported ED symptom was invited to participate, with an original goal of 1000 participants representing the community. There were no exclusion criteria. Participants gave informed consent and could cease or withdraw at any time. The cohort was drawn nationwide with representation from all states and territories, all under restrictions mandated by the Australian Government Department of Health for the duration of the study.

#### Australia’s COVID-19 public health response

From March 2020 with some easement of measures starting in October 2020, Australian borders were closed to all non-residents, with returning residents required to spend two weeks in supervised quarantine hotels, all state and territory borders were closed within Australia, and social distancing rules were in place. After a surge of cases in March 2020, public health measures saw a significant reduction in cases to less than 20 per day by the end of April, and Australia had essentially eradicated the virus by November 2020, excepting quickly contained local outbreaks. As of March 2021, Australia has had 29,000 COVID-19 cases with 909 deaths [26]. Forty-five percent of the study cohort was from the state of Victoria who experienced an additional stay-at-home order July to October 2020 due to an outbreak in May, significant to the survey period.

### Measures

Following demographic (variables benchmarked by the Australian Bureau of Statistics where possible), co-occurring mental illness (confirmed diagnoses, self-reported), ED treatment (e.g. general practitioner, psychologist, dietician, etc.) and items pertaining to the experience of ED prior to the pandemic (*pre-pandemic*), participants were asked to rate any change in ED symptom they had experienced during the pandemic (*within-pandemic*) (i.e., body image concern, food restriction/dieting, binge/over-eating, etc.) and other pandemic experiences (i.e., quality of sleep, alcohol use, etc.) on a 5-point Likert scale (i.e., *increased a lot-decreased a lot*) (Additional file 1). Participants were also asked about the impact of specific pandemic experiences, including public health measures (i.e., *positive, neutral,* or *negative*). As no validated “pandemic impact” validated measure existed at the time, the survey was designed by a collaboration of researchers, clinicians, biostatisticians, and peer researchers with lived experience and approved by ethics.

Participants completed three standardised measures: *The Eating Disorder Examination – Questionnaire 6.0* (EDE-Q) [27], a self-report measure used to assess present-state ED psychopathology; The *Depression Anxiety Stress Scale – 21* (DASS-21) [28], a set of three self-report scales designed to measure the negative emotional states of depression, anxiety and stress; and the *UCLA Loneliness Scale* (Version 3) [29] to assess how often a person feels disconnected from others [30] (Additional file 2 for more detail).

### Statistical analyses

Data were cleaned and recoded for analysis using STATA Version 15 (StataCorp, College Station, TX, USA) and IBM SPSS Version 26. Descriptive statistics summarised the results with contingency tables, measures of central tendency and dispersion. A series of multivariate linear regressions were examined to infer statistical significance of the difference of validated measures between dichotomous ED symptom change. Furthermore, inference of associations was examined with Pearson’s chi squared tests to explore dichotomous variables. An alpha level of 0.05 was used for all statistical tests.

The online survey was widely promoted, and 2326 people consented to the study. However, 474 people were considered non-starters and their missing data was not included in analysis as they discontinued in the early stages of the survey (by question 25 of a 117-question survey) as per the following breakdown: 117 did not complete one question, 69 discontinued within the first five demographic questions, and 288 discontinued prior answering questions relating to impact of the pandemic (question 25, primary outcome variable). In addition, 129 reported no ED diagnosis or symptoms past or present and so did not meet inclusion criteria. Therefore, 1723 people were considered active participants and included in the analysis. Due to direction from the approving Human Research Ethics Committee, all survey questions and formal measures were optional, so the *n* for each response is noted with the reporting of each statistical analysis reported in text, figures, and tables.

## Results

### Demographics

Of the 1723 participants, mode age was 24.9 years (age range collected), range 16–80 years, with 83.1% (*n* = 1431) under 30 years. The majority (97.2%) were assigned the female gender at birth (*n* = 1675, male *n* = 46, prefer not to say *n* = 2), with 91.6% (*n* = 1578) identifying as female. Forty-five participants (2.6%) identified as Aboriginal or Torres Strait Islander. Most participants lived in urban areas (81.5%), over half with their families (55.7%) or partner (16.4%), and 9.3% were parents. The cohort was well educated, with 40.9% having graduated high school and an additional 51.9% higher qualification or degree, and 68.3% were in paid employment prior to the pandemic. Full demographics in Additional file 3. Potential seasonal and time related affects such as the month of completion of the survey were analysed and it was found there was no statistically significant difference (*p* = 0.388) between the completion months on the EDE-Q Global Score.

#### Pre-existing eating disorder illness factors

Thirty-nine percent (*n* = 679) self-reported a current DSM-5 [25] eating disorder (ED) diagnosis, 20.1% (*n* = 346) a previous diagnosis, with 40.5% (*n* = 698) not having ever received formal diagnosis but self-identified ED symptoms. Additional file 3 shows the proportion of the sample by diagnostic group, the largest proportion reporting a lifetime experience of Anorexia Nervosa (AN) (42.3%). To note, participants could identify more than one diagnosis, with 33.1% identifying multiple diagnoses.

This cohort reported significant current eating disorder symptomatology. Of those who completed the EDE-Q (*n* = 1327), the mean EDE-Q Global Score was 4.08 (*SD* = 1.19, range 0.06 to 6.00). Participants were asked to identify active ED symptoms they had been experiencing just prior to the start of the pandemic (i.e., prior to March 1, 2020 labelled *Current*): 90.8% (*n* = 1565) body image concern, 75.5% (*n* = 1300) food restriction/dieting, 55.6% (*n* = 958) over/binge eating, 25.0% (*n* = 430) self-induced vomiting, 37.1% (*n* = 639) driven over-exercise, and 14.9% (*n* = 257) laxative or water pill misuse (multiple responses allowed). Only 100 participants reported diet pill misuse but as reported increase in symptom was low for this group (*n* = 4), no further analysis was interpreted. Full data are presented in Additional file 4.

Participants reported a history of ED symptomatology for a mean (*x*) 9.28 years (*SD* = 8.45, range 1–53 years, Median 7 and Interquartile range 3–12 years). Prior to the pandemic, the vast majority (95.9%) reported experiencing ED symptoms at least some of the time: 20.5% all the time, 45.0% most of the time, 16.4% half of the time, 14.0% some of the time, with 4.1% none of the time (but previously). Nearly half (47.9%) were receiving some form of ED treatment during the pandemic, 25.7% had received treatment in the past, and 26.5% had never received any treatment. To note, there was a significant difference between EDE-Q Global Score for those with a treatment history (*x* = 4.19, *SD* = 1.16) compared to those with none (*x* = 3.78, *SD* = 1.19), *t*(1265) = 5.58, *p* < 0.001. One hundred and twenty-seven participants had an inpatient admission within the past twelve months, and an additional 168 reported an admission prior to that, representing 17.1% of the sample reporting receipt of inpatient care.

#### Pre-existing co-occurring mental health factors

Seventy-nine percent reported a current or lifetime co-occurring mental health condition: 71.1% obsessive–compulsive disorder, 70.9% anxiety, 55.2% depression, and 65.2% a history of self-harm, suicidal ideation or at least one suicide attempt (24.7% currently, 40.5% previously), detailed in Additional file 3 (multiple responses allowed). Considering mental state within the week of baseline reporting, participants reported on average severe depression (*x* = 24.72, *SD* = 11.96), severe anxiety (*x* = 17.72, *SD* = 10.34) and moderate stress (*x* = 23.98, *SD* = 10.06) on the Depression Anxiety Stress Scale (DASS21) and elevated feelings of loneliness (UCLA Loneliness Scale: *x* = 54.54, *SD* = 12.10, range 2–80 with a maximum of 80).

#### Impact of COVID-19

Participants were asked about the direct impact of COVID-19 on themselves or an immediate family member: 629 self-quarantined after being a close contact with an identified case, with 108 of that group falling physically ill with the virus. An additional 65 people were hospitalised, with 20 of those passing away. To note, 107 participants declined to answer. Participants rated feelings related to their experience of the pandemic overall on a visual analogue scale: Worry *x* = 38.93 [0 (very worried)—100 (not worried), *SD* = 24.94]; Fear *x* = 44.41 [0 (very fearful)—100 (not fearful), *SD* = 25.23]; Confidence *x* = 41.91 [0 (not at all confident) – 100 (very confident), *SD* = 22.15]; and Hopefulness *x* = 43.25 [0 (not at all hopeful) – 100 (very hopeful), *SD* = 22.98], although wide variance is noted. Participants with higher scores on the EDE-Q Global Score, DASS and UCLA Loneliness, were significantly more likely to report more worry and fear, and less confidence and hopefulness, relating to their pandemic experience (Table 1).

**Table 1:** Changes in eating disorder symptoms and impact on mental health during the pandemic

Eating disorder symptom	Formal measure	*n*	Coefficient	SE	*t*	95% confidence interval	*p*
Body image concern	EDEQ-GS	1327	0.96	0.10	9.77	**(0.77, 1.15)**	**< .001**
	DASS Depression	1218	3.59	1.07	3.35	**(1.49, 5.69)**	**.001**
	DASS Depression	1191	2.53	0.93	2.72	**(0.70, 4.35)**	**.007**
	DASS Depression	1239	2.97	0.88	3.39	**(1.25, 4.69)**	**.001**
	UCLA Loneliness	1233	2.68	1.06	2.53	**(0.60, 4.76)**	**.012**
Food restriction/dieting	EDEQ-GS	1327	0.61	0.07	8.37	**(0.47, 0.76)**	**< .001**
	DASS Depression	1218	2.62	0.79	3.33	**(1.07, 4.16)**	**.001**
	DASS Depression	1191	2.79	0.67	4.14	**(1.47, 4.11)**	**< .001**
	DASS Depression	1239	3.39	0.65	5.23	**(2.11, 4.66)**	**< .001**
	UCLA Loneliness	1233	2.06	0.79	2.62	**(0.52, 3.61)**	**.009**
Binge/over-eating	EDEQ-GS	1327	0.23	0.07	3.41	**(0.10, 0.36)**	**.001**
	DASS Depression	1218	1.52	0.71	2.15	**(0.13, 2.92)**	**.032**
	DASS Depression	1191	− 0.46	0.61	− 0.76	(− 1.66, 0.73)	.448
	DASS Depression	1239	0.38	0.59	0.64	(− 0.78, 1.54)	.524
	UCLA Loneliness	1233	1.66	0.71	2.32	**(0.26, 3.06)**	**.02**
Self-induced vomiting	EDEQ-GS	1327	0.65	0.07	8.80	**(0.51, 0.80)**	**< .001**
	DASS Depression	1218	4.49	0.78	5.74	**(2.95, 6.02)**	**< .001**
	DASS Depression	1191	4.03	0.67	6.02	**(2.72, 5.34)**	**< .001**
	DASS Depression	1239	2.68	0.66	4.07	**(1.39, 3.97)**	**< .001**
	UCLA Loneliness	1233	1.88	0.80	2.35	**(0.31, 3.45)**	**.019**
Driven/over-exercise	EDEQ-GS	1327	0.44	0.06	6.81	**(0.31, 0.56)**	**< .001**
	DASS Depression	1218	0.20	0.68	0.30	(− 1.14, 1.54)	.767
	DASS Depression	1191	1.74	0.58	2.98	**(0.60, 2.89)**	**.003**
	DASS Depression	1239	2.45	0.57	4.33	**(1.34, 3.56)**	**< .001**
	UCLA Loneliness	1233	0.75	0.69	1.09	(− 0.60, 2.10)	.276
Laxative and/or pill misuse	EDEQ-GS	1327	0.64	0.09	6.89	**(0.46, 0.82)**	**< .001**
	DASS Depression	1218	3.93	0.97	4.05	**(2.03, 5.84)**	**< .001**
	DASS Depression	1191	4.06	0.83	4.89	**(2.43, 5.69)**	**< .001**
	DASS Depression	1239	2.73	0.81	3.36	**(1.14, 4.33)**	**.001**
	UCLA Loneliness	1233	5.75	0.97	5.90	**(3.84, 7.66)**	**< .001**
Diet pill misuse	EDEQ-GS	1327	0.35	0.59	0.59	(− 0.81, 1.51)	.555
	DASS Depression	1218	5.03	5.93	0.85	(− 6.61, 16.67)	.397
	DASS Depression	1191	7.73	5.04	1.53	(− 2.16, 17.62)	.125
	DASS Depression	1239	0.02	5.00	0.00	(− 9.79, 9.83)	.997
	UCLA Loneliness	1233	4.08	6.02	0.68	(− 7.73, 15.90)	.498

Pandemic experience	Formal measure	*n*	Coefficient	SE	*t*	95% confidence interval	*p*
Worried	EDEQ-GS	1326	− 0.18	0.07	− 2.52	**(− 0.32, − 0.04)**	**.012**
	DASS Depression	1217	− 2.92	0.75	− 3.88	**(− 4.39, − 1.44)**	**< .001**
	DASS Depression	1190	− 3.56	0.64	− 5.54	**(− 4.82, − 2.30)**	**< .001**
	DASS Depression	1238	− 2.81	0.62	− 4.49	**(− 4.03, − 1.58)**	**< .001**
	UCLA Loneliness	1232	− 2.44	0.76	− 3.22	**(− 3.92, − 0.95)**	**.001**
Fearful	EDEQ-GS	1326	− 0.18	0.07	− 2.68	**(− 0.31, − 0.05)**	**.007**
	DASS Depression	1217	− 2.48	0.70	− 3.56	**(− 3.85, − 1.11)**	**< .001**
	DASS Depression	1190	− 3.40	0.60	− 5.71	**(− 4.57, − 2.24)**	**< .001**
	DASS Depression	1238	− 2.38	0.58	− 4.09	**(− 3.51, − 1.24)**	**< .001**
	UCLA Loneliness	1232	− 2.08	0.70	− 2.97	**(− 3.46, − 0.71)**	**.003**
Confident	EDEQ-GS	1326	− 0.36	0.07	− 5.54	**(− 0.49, − 0.24)**	**< .001**
	DASS Depression	1217	− 5.25	0.68	− 7.73	**(− 6.58, − 3.92)**	**< .001**
	DASS Depression	1190	− 3.89	0.59	− 6.61	**(− 5.04, − 2.73)**	**< .001**
	DASS Depression	1238	− 4.40	0.57	− 7.76	**(− 5.51, − 3.29)**	**< .001**
	UCLA Loneliness	1232	− 4.60	0.69	− 6.68	**(− 5.95, − 3.25)**	**< .001**
Hopeful	EDEQ-GS	1326	− 0.27	0.07	− 4.19	**(− 0.40, − 0.15)**	**< .001**
	DASS Depression	1217	− 6.06	0.67	− 9.09	**(− 7.36, − 4.75)**	**< .001**
	DASS Depression	1190	− 3.86	0.58	− 6.64	**(− 5.00, − 2.72)**	**< .001**
	DASS Depression	1238	− 3.59	0.57	− 6.36	**(− 4.70, − 2.48)**	**< .001**
	UCLA Loneliness	1232	− 4.33	0.68	− 6.34	**(− 5.67, − 2.99)**	**< .001**
Victorian resident	EDEQ -GS	1327	− 0.03	0.07	− 0.41	(− 0.16, 0.10)	.679
	DASS Depression	1218	1.39	0.68	2.03	**(0.05, 2.72)**	**.042**
	DASS Depression	1191	− 0.87	0.59	− 1.49	(− 2.03, 0.28)	.137
	DASS Depression	1239	0.28	0.57	0.49	(− 0.84, 1.40)	.624
	UCLA Loneliness	1233	0.37	0.69	0.53	(− 0.98, 1.72)	.595

Bold indicates statistical significance

n = subsample size; SE = standard error; CI = confidence interval; p = p-value significant at p < .05; t = t-statistic; EDEQ GS = Eating Disorder Examination Questionnaire Global Score; DASS = Depression, Anxiety, Stress Score; UCLA Loneliness = University of California, Los Angeles Loneliness Scale

#### Impact of COVID-19 on eating disorder symptoms

**Fig. 1:**
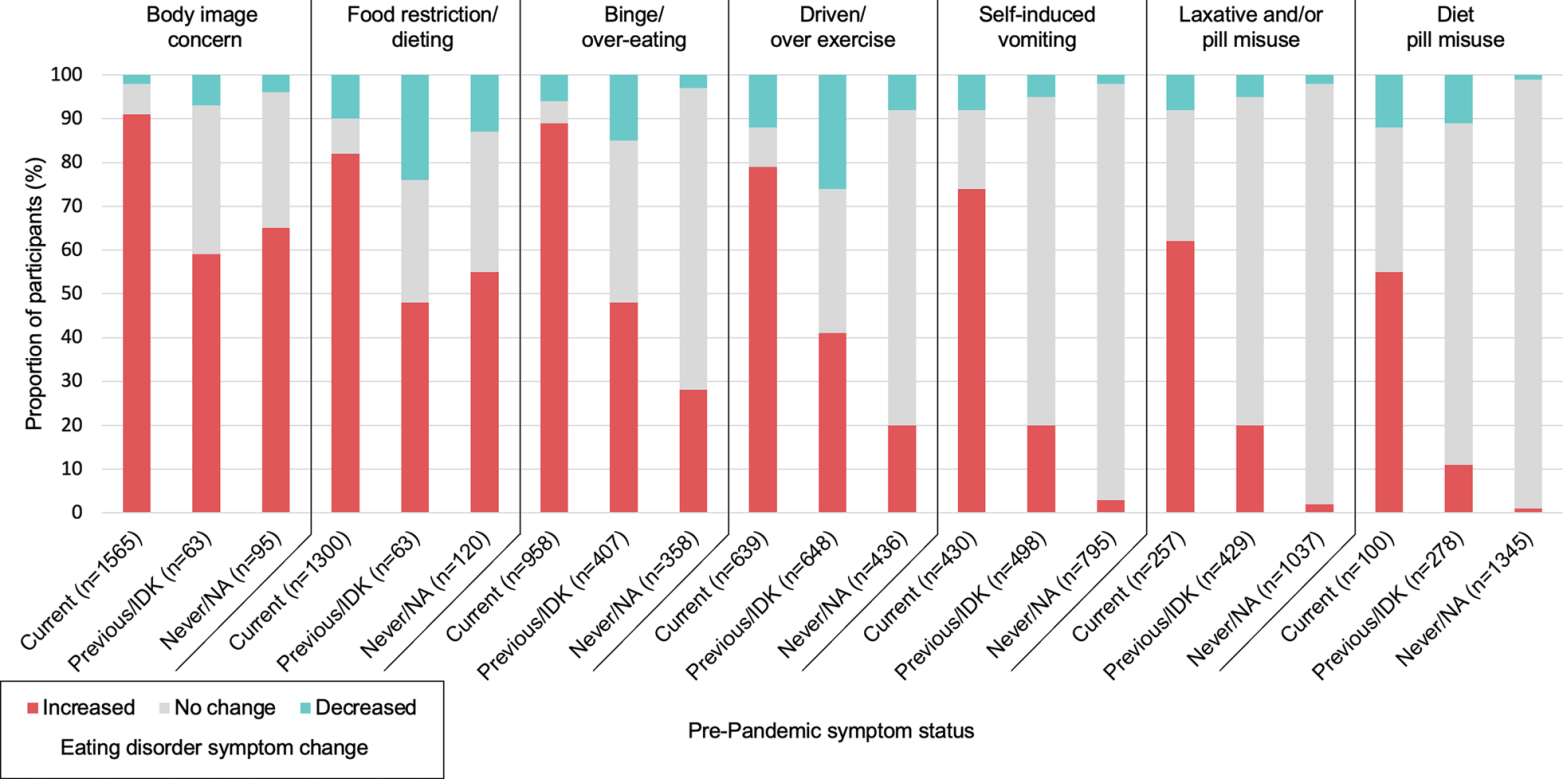
Changes in eating disorder symptoms during the pandemic compared to pre-pandemic symptom status. *IDK* I don’t know (i.e., if symptom was of note), *NA* not applicable

Participants were asked to rate degree of change of ED symptoms at assessment (*within pandemic*) compared to before the pandemic (*pre-pandemic*). Presented in Fig. 1 (and Additional file 4) are responses by the total cohort and by subgroups dependent on whether the ED symptom was present pre-pandemic or not. As can be seen overall, there is a reported increase across most ED symptoms within pandemic: 88.0% of the total sample reported an increase in body image concern (67.2% a lot, 20.8% somewhat), 74.1% in food restriction/dieting, 66.2% in binge/over-eating, 48.6% in driven/over-exercise and 25.4% in self-induced vomiting. Of the 1565 who reported body image concern as a current symptom prior to the pandemic, 90.5% reported an increase (70.3% a lot, 20.2% somewhat), of the 1300 who reported food restriction/dieting as a current ED symptom prior to the pandemic, 82.1% reported an increase, and of the 958 who reported binge/over-eating as a current ED symptom prior to the pandemic, 89.2% reported an increase.

Although applying to much smaller proportions of the cohort, reporting re-emergence and first-time emergence of ED symptoms during the pandemic was not uncommon. As seen in Fig. 1, 59.3% of participants reporting previous (i.e., a lifetime ED symptom that was not active prior to the pandemic) body image concerns reported symptom return during pandemic. Similarly, 47.7% who reported lifetime food restriction/dieting, 47.2% binge/over-eating and 40.5% driven/over-exercise reported re-emergence of those symptoms. For some participants who reported they had never experienced (or the symptom was *not applicable*) a particular ED symptom prior to the pandemic, they experienced it for the first time during the pandemic: 65.3% reporting increased body image concerns; 37.5% food restriction/dieting, and 26.8% binge/over-eating.

Multivariate linear regressions were used to explore the relationship between change in ED symptoms reported during the pandemic with ED severity adjusting for demographics as potential confounders. For all ED symptoms, participants who reported an increase in ED symptoms during the pandemic (*within pandemic*) were significantly more likely to have a higher eating disorder severity score as indexed by the EDE-Q Global Score, for example, body image concern (Coefficient = 0.96; *p* < 0.001, 95% CI 0.77 to 1.15) and food restriction/dieting (Coefficient = 0.61; *p* < 0.001, 95% CI 0.47 to 0.76) (Table 1).

Pearson’s chi-square test of independence with corresponding relative risk (*RR*) and confidence intervals (*CI*) were performed to explore relationships between change in ED symptoms within pandemic with ED diagnostic status and ED treatment status (Table 2). The relationships between ED symptom and presence of ED diagnosis were mixed, for example, participants with current ED diagnosis (39.4%) were 1.6 times more likely to report an increase in self-induced vomiting during the pandemic as those without diagnosis (*RR* 1.62; 95% CI 1.442 to 1.811), but 25% less likely to report an increase in binge/over-eating (*RR* 0.75; 95%*CI*, 0.667 to 0.842). The relationship between ED treatment status (i.e., whether participants were engaged in active treatment or not) and ED symptom change were also mixed. The 47.9% of the sample engaged in ED treatment were more likely to report an increase in self-induced vomiting (1.3 times), laxative and/or pill misuse (1.4 times) and driven/over-exercise (1.2 times), but 22% less likely to report an increase in binge/over-eating.

**Table 2:** Changes in eating disorder symptoms by diagnostic status, presence of co-occurring conditions, engagement in treatment and residing in the state of Victoria during the pandemic

	Eating disorder diagnosis				Co-occurring condition				Receiving eating disorder treatment				Victorian resident			
	Diagnosis	No diagnosis	*χ*^2^ (*p*)	*RR* (95%*CI*)	Co-occurring condition	No co-occurring condition	*χ*^2^ (*p*)	*RR* (95%*CI*)	Receiving treatment	Not receiving treatment	*χ*^2^ (*p*)	*RR* (95%*CI*)	Yes	No	*χ*^2^ (*p*)	*RR* (95%*CI*)
Body image concern																
Increased	594	923	0.20 (.655)	0.96 (0.805, 1,145)	1206	311	**7.13 (.008)**	**1.11 (1.018, 1.219)**	728	789	0.06 (.808)	1.02 (0.874, 1.189)	711	806	3.64 (.056)	1.18 (0.987, 1.404)
Not increased	84	122			147	59			97	109			82	124		
Food restriction/ dieting																
Increased	525	751	**6.64 (.010)**	**1.20 (1.041, 1.389)**	1019	257	**5.18 (.023)**	**1.07 (1.006, 1.135)**	627	649	3.11 (.078)	1.11 (0.986, 1.248)	615	661	**9.35 (.002)**	**1.21 (1.066, 1.375)**
Not increased	153	294			334	113			198	249			178	269		
Binge/over-eating																
Increased	403	737	**22.58 (< .001)**	**0.75 (0.667, 0.842)**	894	246	0.22 (.882)	1.00 (0.946, 1.049)	497	643	**24.79 (< .001)**	**0.78 (0.703, 0.854)**	535	605	1.11 (.292)	1.06 (0.952, 1.184)
Not increased	275	308			459	124			328	255			258	325		
Self-induced vomiting																
Increased	237	193	**59.68 (< .001)**	**1.62 (1.442, 1.811)**	341	89	0.21 (.651)	1.01 (0.958, 1.072)	254	176	**28.74 (< .001)**	**1.34 (1.211, 1.478)**	181	249	3.57 (.059)	0.889 (0.785, 1.008)
Not increased	441	852			1012	281			571	722			612	681		
Driven/over exercise																
Increased	347	476	**5.22 (.022)**	**1.15 (1.020, 1.289)**	654	169	0.83 (.364)	1.02 (0.974, 1.075)	427	396	**10.11 (.001)**	**1.17 (1.063, 1.295)**	395	428	2.46 (.117)	1.09 (0.980, 1.202)
Not increased	331	569			699	201			398	502			398	502		
Laxative and/or pill misuse																
Increased	131	109	**27.11 (< .001)**	**1.48 (1.295, 1.691)**	200	40	3.82 (.051)	1.07 (1.007, 1.141)	148	92	**21.23 (< .001)**	**1.35 (1.205, 1.514)**	100	140	2.13 (.144)	0.89 (0.760, 1.046)
Not increased	547	936			1153	330			677	806			693	790		
Diet pill misuse^a^																
Increased	4	1	3.47 (.062)	2.04 (1.310, 3.173)	5	0	1.37 (.242)	1.27 (1.243, 1.306)	4	1	2.07 (.150)	1.67 (1.077, 2.602)	2	3	0.07 (.787)	0.87 (0.297, 2.545)
Not increased	674	1044			1348	370			821	897			791	927		

Bold indicates statistical significance

*n* = sample size; χ^2^ = Chi-square Statistic; *p* = p-value significant at *p* < .05; *CI* = Confidence Interval; *RR* = Relative Risk; *t* = t-statistic; *df* = degrees of freedom ^@a^Number of participants reporting diet pill misuse as a symptom was very low so analysis unreliable and no interpretation drawn

#### Relationship of mental state to eating disorder symptom change

Multivariate linear regressions were used to explore the relationship between ED symptom change during the pandemic with mental state (DASS-21) and loneliness (UCLA Loneliness Scale) (Table 1). Higher DASS depression scores were associated with an increase in most ED symptoms within pandemic, such as for body image concern (Coefficient = 3.59; *p* = 0.001, 95% CI 1.49–5.69), food restriction/dieting (Coefficient = 2.62; *p* = 0.001, 95% CI 1.07–4.16), and self-induced vomiting (Coefficient = 4.49; *p* < 0.001, 95% CI 2.95–6.02) with a moderate to high effect size. Higher DASS anxiety and stress scores were significantly associated with increased body image concern, food restriction/dieting, self-induced vomiting, driven/over-exercise and laxative and/or pill misuse. Finally, higher loneliness scores were associated with an increase in all ED symptoms except for driven/over-exercise.

Pearson’s chi-square test of independence with corresponding relative risks and confidence intervals were performed to examine the relation between the presence of at least one co-occurring mental health condition (79.0%) and change in ED symptom during the pandemic (Table 2). Participants who self-reported a current co-occurring mental health condition were 1.1 times more likely to experience an increase in body image concern (*RR* 1.11; 95% *CI* 1.018–1.219) and food restrictions/dieting (*RR* 1.07; 95% *CI* 1.006–1.135) within pandemic, noting modest effect sizes. Regarding the impact of the pandemic on other aspects of mental health: 87.5% of participants reported guilt over buying food, 69.3% a poorer quality of sleep, 50.3% increased alcohol use, 34.5% increased use of prescription medication, 32.3% increased smoking and 31.7% increased recreational drug use.

#### Impact of public health measures

Presented in Table 3, most participants were negatively impacted by a variety of public health measures and pandemic experiences, for example, 83.0% were negatively impacted from change in daily routine, 81.0% from restricted access to family and friends, 68.0% from social media reaction to the pandemic and 62.0% from news coverage. Multivariate linear regressions were used to explore the relationship between eating disorder severity as indexed by the EDE-Q Global Score and whether these experiences caused a negative impact, as opposed to a neutral/positive impact. There was a significant relationship between higher EDE-Q Global scores in 12 out of 17 experiences, such as promotion of exercise as an essential activity (Coefficient = 0.53, *p* < 0.001, 95% CI 0.41–0.65), change in daily routine (Coefficient = 0.48; *p* < 0.001, 95% CI 0.32–0.65), availability of safe foods on meal plan, increased focus on cleaning/hygiene, and change in access to health professionals/treatment, to name a few.

**Table 3:**
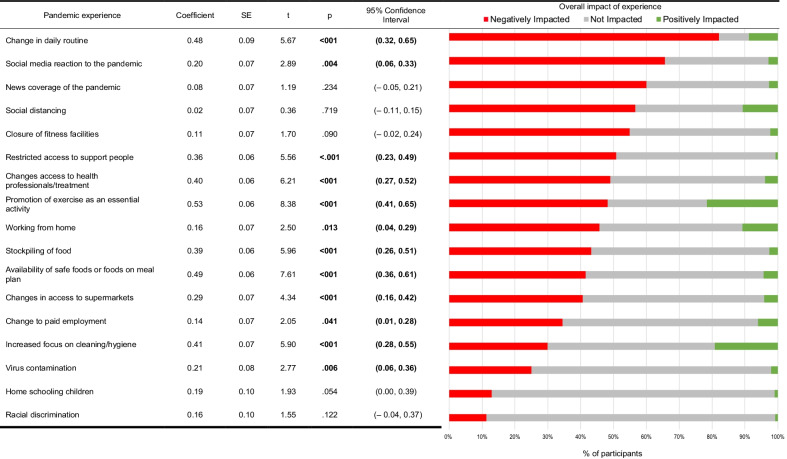
Pandemic experience and impact on eating disorder index (*N* = 1723)

N, sample size; SE, standard error; CI, confidence interval; *p*, *p*-value significant at *p* < .05; t*,* t-statistic

Residents of the state of Victoria (45.6%) experienced stricter public health measures for most of the study duration so a comparison of impact was made to the rest of Australia. Current ED illness level as indexed by EDE-Q Global score (Coefficient = − 0.03; *p* = 0.679, 95% CI − 0.16 to 0.10) was not significantly different for Victorian residents (Table 1), and the only significant ED symptom increase was for food restrictions/dieting behaviour (*RR* 1.2; 95%*CI*, 1.07 to 1.38) (Table 2). While there was no significant difference in stress, anxiety or loneliness, Victorian participants reported significant difference from the rest of Australia for depression (Coefficient = 1.39; *p* = 0.042, 95% CI 0.05–2.72) to a large effect size.

## Discussion

This work represents one of the only national studies to have captured the impact of the pandemic on this pervasive and sometimes life-threatening psychiatric disorder during the height of a unique global experience. This was a well-advertised online community observational survey inviting anyone with a lived experience of an eating disorder to participate with a large response rate of over 1700 participants compared to other published studied to date. As might be expected for this population, 91% of the cohort identified as female. The cohort was overall skewed younger (mode 24.9 years) than other community studies [31], potentially due to the online nature and social media promotion of the study. Forty percent had never sought formal assessment for an eating disorder and 27% had never sought treatment, highlighting the importance of broad community-based recruitment for research to access those not engaged in the health system [32]. The community cohort reported a high level of ED symptomatology with mean EDE-Q global score comparable to expected norms for a clinical population (*x* = 4.02, *SD* = 1.28) and distinct from a non-clinical population (*x* = 0.93, *SD* = 0.86), [32], with Anorexia Nervosa (17.1% current diagnosis, 19.4% previous diagnosis) being the most prevalent diagnostic group and body image concerns (90.8%) and food restriction (75.5%) the most pervasive symptoms.

As with emerging research [19, 23, 33], the hypothesis of the detrimental impact of the pandemic on the symptomatology of people with a lived experience of an eating disorder was well supported here, with 88.0% of the total cohort reporting increase in body image concern, 74.1% an increase in food restriction/dieting, 66.2% in binge/over-eating, 48.6% in driven/over-exercise and 25.4% in self-induced vomiting. For those reporting an ED symptom present at pandemic onset, a stronger impact of the pandemic was found across all ED symptoms, with 90.5% of participants who reported body image concern prior to the pandemic reporting an increase during the pandemic through to 54.6% for laxative and/or pill misuse. For the subset of participants who reported resolved ED symptoms pre-pandemic, nearly half reported symptoms re-emerging during the pandemic, namely body image concern (for 59.3% of the subset), food restriction/dieting (47.7%), binge/over-eating (47.2%), and driven/over exercise (40.5%), similar to reports of 41.9% of patients being treated for an ED experiencing a reactivation of symptoms [21]. There was a subset of people in every ED behaviour category that reported first-time emergence of that symptom during the pandemic, such as body image concern (62 out of 95 participants), food restriction/dieting (65/120), and binge/over-eating (96/358) (Additional file 4), although these subsets were small compared to the proportion of the sample reporting a current experience of each symptom.

Overall, findings of global increase across all ED symptoms, whether present prior to the onset of the pandemic or not, are of clinical concern, particularly noting that over half of the cohort were not engaged in any form of treatment during the pandemic. The pandemic impacted those with more severe ED illness more significantly than those with less severe illness (Table 1), with increase in all ED symptoms associated with higher EDE-Q scores and/or formal diagnosis (except for binge/over-eating). Current treatment engagement related to less likely reporting of increased binge/over-eating but an increase in self-induced vomiting, driven/over exercise, and laxative misuse, however those engaged in treatment did report more severe illness and likely more impact on symptoms due to the pandemic. Ongoing data collection to 6 months follow up, including analysis of the relationship of type and intensity of treatment during the pandemic, particularly the role of telehealth, is ongoing and will provide insights into the effect of treatment on symptoms during the pandemic.

Co-occurring mental health conditions are prevalent in ED cohorts and evidence suggests can increase the risk of severe eating disorder psychopathology [34]. The cohort reported 79% having at least one co-occurring mental health condition and the presence of at least one co-occurring condition was associated with increased body image concern and food restriction during the pandemic, with higher mental health states of depression, anxiety and stress significantly associated with an increase in most ED symptoms. Social isolation and loneliness are risk factor common to EDs [16] and has been predictive of general psychiatric disorders during the pandemic especially in young women [35], a group in which EDs are common; in this study, higher reported loneliness was found to be significantly associated with all ED symptoms except for driven/over-exercise. Other indicators of declining mental health were reported, such as reduced sleep quality (69.3%) and increased alcohol use (50.3%). Sixty five percent of participants reported a history of self-harm and/or suicidal ideation or attempt, including a quarter in the 12 months prior to the pandemic. Given the association between EDs and self-harming behaviours [36] and escalating suicide risk due to the profound psychological and social effects of the pandemic in general [37], people with a lived experience of an eating disorder need to be particularly aware of the risk of declining mental health during such times of stress and uncertainty.

Even though only 10% of the sample reported a direct impact of the COVID-19 virus, namely themselves or an immediate family member becoming unwell, most reported high levels of worry and fear, and low levels of confidence and hopefulness associated with the pandemic. To curb viral spread, Australia’s public health response was profound, and the impact of public health measures and general pandemic experiences had a negative impact for most participants, more significantly for those with more acute ED illness, such as the impact of changes in daily routine, social media, and news coverage of the pandemic, and reduced access to social supports and health professionals and treatments. Negative impact of the intensity of public health measures (measured by the comparison of the participants residing in the state of Victoria to the rest of Australia) was only found for an increase in food restriction/dieting and a higher level of state depression. The overall public health response in Australia was timely, consistent, and comprehensive, so increased restrictions for Victorian residents 5 months into the pandemic may have had minimal negative impact for study participants. In Australia, the pandemic itself has had a comparably mild impact compared to international standards yet a profound one for people with a lived experience of EDs, so it stands to reason that this population may be more at risk in those countries where the pandemic has more devastating repercussions.

Given the necessity for social distancing, online data collection was essential and therefore the self-report nature of observations without clinical validation of ED diagnosis or symptom severity is noted. A self-selected sample is open to selection bias, including only those who are motivated to participate in research. Another limitation was asking people retrospectively about their symptoms prior to the pandemic (pre-pandemic) and then at the time of the survey (within pandemic). While noted this is not as ideal as having baseline data collected and retrospective recall bias may impact data, a large community cohort was not already engaged in eating disorder research prior to the onset of the pandemic that could have been utilised. Participants were asked directly about a range of symptoms as a validated measure did not exist at the time to access change due to the pandemic. However, high quality validated instruments with demonstrated good concordance with clinical presentation were used and the elevated ED index indicates this sample as indicative of a clinical population consistent with previous studies reporting high levels of body shape and weight concerns and eating disorder symptomatology in the community [38]. Due to the rapid need for data collection within a critical timeframe, this study required a prerequisite of written and computer literacy in the English language which may have limited demographic diversity.

## Conclusion

The Coronavirus pandemic has had a profound impact on the global community, with varied but concerted efforts to protect those most vulnerable from the infectious disease. Comprehensive public health response has flattened the viral spread curve, however the World Health Organization warn of an accompanying mental health pandemic for generations to come requiring concerted action from Governments, communities and at risk individuals [6]. The current study presents the impact of COVID-19 pandemic on people with a lived experience of an eating disorder within the Australian population to highlight the risk, especially for those with higher severity of illness, those with co-occurring mental health conditions, and those not currently engaged in clinical care, which was over half of our sample. Only 25% of people actively seek treatment for an ED [4] with up to 70% never accessing any care [39], and, as in other mental illness, lack of contact with the health care system is a risk factor for deterioration, the development of chronic illness, and death [40]. Previous research have suggested eating disorder assessment and management is challenged within primary health care settings [12], so these findings highlight the importance of regular comprehensive mental health assessment and treatment across the health care system, especially for eating disorders, across the community presenting for care during this critical time. Increased awareness, screening, assessment, and an increase in the available options for treatment may well be needed to deal with the effects of the pandemic on this illness group and mental health more broadly.

## Supplementary Information

**Additional file 1.** COVID19 Impact Survey (extract).

**Additional file 2.** Standardised measures.

**Additional file 3.** Participant demographics and illness characteristics.

**Additional file 4.** Changes in eating disorder symptoms during the pandemic.

## Data Availability

The data that support the findings of this study are available on request from the corresponding author. The data are not publicly available due to privacy and ethical restrictions.
