# Supplementary material for: The impact of the COVID-19 pandemic and associated public health response on people with eating disorder symptomatology: an Australian study
Source: J Eat Disord. 2022 Jan 17;10:9. doi: 10.1186/s40337-021-00527-0 (PMC8762631; doi:10.1186/s40337-021-00527-0)
Supplement: Supplementary file 1 — Additional file 1. COVID19 Impact Survey (extract). [file 40337_2021_527_MOESM1_ESM.docx]

***The impact of the COVID-19 pandemic and associated public health response on people with eating disorder symptomatology: An Australian study***

**Supplementary item 1:** COVID19 Impact Survey (extract)

**This section asks for information about your experience with an eating disorder prior to March 1, 2020, prior to the COVID-19 pandemic:**

1. Have you ever received any of the following eating disorder diagnoses?

(For all: Currently – Previously – Never – I don’t know - NA)

- 1. Anorexia Nervosa
  2. Bulimia Nervosa
  3. Binge Eating Disorder
  4. Other or Unspecified Feeding or Eating Disorder
  5. Unsure of specific diagnosis
  6. No

1. If you have not been formally diagnosed with an eating disorder, have you ever experienced any of the following symptoms:

(For all: Currently – Previously – Never – I don’t know - NA)

- 1. Food restriction/dieting
  2. Over/binge eating
  3. Self-induced vomiting
  4. Driven over exercise
  5. Laxative and/or diuretic misuse
  6. Diet pill misuse
  7. Body image concern
  8. Other (free text)

**The next section asks for information about your current experience with an eating disorder since March 1, 2020, after the start of the COVID-19 pandemic:**

1. Since the start of the COVID-19 pandemic, have you experienced a change in any of the following:

(For all: Increased a lot, Increased somewhat, No change, Decreased somewhat, Decreased a lot, NA)

- 1. Food restriction/dieting
  2. Over/binge eating
  3. Self-induced vomiting
  4. Driven over exercise
  5. Laxative and/or diuretic misuse
  6. Diet pill misuse
  7. Body image concern
  8. Guilt about buying food
     Alcohol use
  9. Smoking
  10. Recreational drug use
  11. Prescription medication use
  12. Quality of sleep

1. How have the following factors associated with the COVID-19 pandemic impacted you over the past month:

(VAS: Negatively impacted - Positively impacted, NA):

- 1. Unpredictable food supply
  2. Changes in access to the supermarket
  3. Availability of “safe” foods or foods on your meal plan
  4. Stockpiling of food
  5. Promotion of exercise as an essential activity
  6. Closure of fitness facilities
  7. Changed access to health professionals/treatment
  8. Change in daily routine
  9. Working from home
  10. Change to paid employment
  11. Home schooling children
  12. Restricted access to support people
  13. Restricted access to family/friends
  14. Increased focus on cleaning/hygiene
  15. Virus contamination
  16. Social distancing
  17. Racial discrimination
  18. News coverage of the pandemic
  19. Social media reaction to the pandemic
