# Supplementary material for: The impact of the COVID-19 pandemic and associated public health response on people with eating disorder symptomatology: an Australian study
Source: J Eat Disord. 2022 Jan 17;10:9. doi: 10.1186/s40337-021-00527-0 (PMC8762631; doi:10.1186/s40337-021-00527-0)
Supplement: Supplementary file 2 — Additional file 2. Standardised measures. [file 40337_2021_527_MOESM2_ESM.docx]

***The impact of the COVID-19 pandemic and associated public health response on people with eating disorder symptomatology: An Australian study***

**Supplementary item 2**: Standardised measures

Eating Disorder Examination – Questionnaire (EDE-Q)

The EDE-Q 6.0 (Fairburn & Beglin, 2008) is a self-report measure that was adapted from the Eating Disorder Examination (Fairburn, Cooper & Connor, 1993). It will be used to assess self-reported eating disorder psychopathology. It is a measure of present state, and, except for the diagnostic items, it is exclusively concerned with the preceding four weeks. It assesses both the frequency of key behaviours (including various forms of overeating and purging) and the severity of psychopathology along certain dimensions (dietary restraint, concern about eating, concern about shape, and concern about weight). It is the gold standard for assessment of eating disorder symptomatology and currently used to assess suitability for the Medicare Benefits Scheme in Australia.

Depression Anxiety Stress Scale – 21 (DASS-21)

The DASS (Lovibond & Lovibond, 1995) is a set of three self-report scales designed to measure the negative emotional states of depression, anxiety and stress. The DASS was constructed not merely as another set of scales to measure conventionally defined emotional states, but to further the process of defining, understanding, and measuring the ubiquitous and clinically significant emotional states usually described as depression, anxiety and stress. The DASS should thus meet the requirements of both researchers and scientist-professional clinicians.

UCLA Loneliness Scale (Version 3)

Developed by psychologist Daniel Russell (1996), the UCLA Loneliness Scale (Version 3) is a 20-item measure that assesses how often a person feels disconnected from others. The measure has been found to be highly reliable in terms of internal consistency and test-retest reliability, convergent validity is indicated by significant correlations with other measures of loneliness, and construct validity is supported by significant relations with measures of the adequacy of the individual's interpersonal relationships and correlations between loneliness and measures of health and well-being (Derrick & White, 2017).

Fairburn CG, Cooper Z, O’Connor M. The eating disorder examination. International Journal of Eating Disorders. 1993;6:1-8.

Fairburn CG, Beglin SJ. Eating disorder examination questionnaire. Cognitive behavior therapy and eating disorders. 2008 Sep:309-13.

Lovibond, S.H. & Lovibond, P.F. (1995).  Manual for the Depression Anxiety Stress Scales. (2nd. Ed.)  Sydney: Psychology Foundation.

Russell, D. W. (1996). UCLA Loneliness Scale (Version 3): Reliability, validity, and factor structure. *Journal of Personality Assessment, 66*(1), 20–40.

Derrick B, White P. Comparing two samples from an individual Likert question. Int J Mathematics Stat. 2017;18(3):1–13.
