# Supplementary material for: The impact of the COVID-19 pandemic and associated public health response on people with eating disorder symptomatology: an Australian study
Source: J Eat Disord. 2022 Jan 17;10:9. doi: 10.1186/s40337-021-00527-0 (PMC8762631; doi:10.1186/s40337-021-00527-0)
Supplement: Supplementary file 3 — Additional file 3. Participant demographics and illness characteristics. [file 40337_2021_527_MOESM3_ESM.docx]

***The impact of the COVID-19 pandemic and associated public health response on people with eating disorder symptomatology: An Australian study***

| **Supplementary item 3.** Participant demographics and illness characteristics (*N*=1723) | | | | |
| --- | --- | --- | --- | --- |
| Category | *n (%)* | Category | | *n (%)* |
| ***Age Group*** *(year range)* |  | ***Location (Territorial Classification)*** | |  |
| 16-20 | 747 (43.4%) | Urban | | 1404 (81.5%) |
| 21-30 | 684 (39.7%) | Regional | | 191 (11.1%) |
| 31-40 | 184 (10.7%) | Rural | | 127 (7.4%) |
| 41-50 | 62 (3.6%) | ***Living Situation*** | |  |
| 51-60 | 34 (2.0%) | I live with my family (including children) | | 959 (55.7%) |
| 61-70 | 9 (0.5%) | I live with my partner/spouse | | 283 (16.4%) |
| 71+ | 3 (0.2%) | I live in a share house | | 262 (15.2%) |
| ***Gender Identified*** |  | I live alone | | 178 (10.3%) |
| Female | 1578 (91.6%) | I live at school/university accommodation | | 21 (1.2%) |
| Non-binary | 81 (4.7%) | I live in supported accommodation | | 16 (0.9%) |
| Male | 42 (2.4%) | I live with my grandparents as a carer | | 3 (0.2%) |
| Transgender Male | 12 (0.7%) | I am homeless | | 1 (0.1%) |
| Transgender Female | 3 (0.2%) | ***Carer Status*** | |  |
| Gender Fluid | 4 (0.2%) | Parent/Guardian | | 160 (9.3%) |
| Agender | 3 (0.2%) | Carer for someone with mental/physical health condition, or an older adult | | 97 (5.6%) |
| ***Ethnicity***^a^ |  | ***Highest qualification*** | |  |
| *Oceanian* |  | Primary School | | 125 (7.3%) |
| Aboriginal/Torres Strait Islander | 45 (2.6%) | High School | | 704 (40.9%) |
| Australian | 862 (50.0%) | Trade certificate, diploma, or apprenticeship | | 259 (15.0%) |
| New Zealander/Maori | 14 (0.8%) | University postgraduate qualification | | 227 (13.2%) |
| Other Oceanian | 2 (0.1%) | University undergraduate qualification | | 408 (23.7%) |
| *African* |  | ***Educational Status*** | |  |
| North African/Middle Eastern | 10 (0.6%) | Studying or enrolled in study prior to the pandemic | | 1076 (62.5%) |
| Sub-Saharan | 8 (0.5%) | ***Employment Status*** | |  |
| *American* |  | In paid employment prior to the pandemic | | 1176 (68.3%) |
| People of the North Americas | 24 (1.4%) | ***Eating Disorder Diagnosis***^b^ | | *n (%)*^c^ |
| People of the South Americas | 8 (0.5%) | Anorexia Nervosa (*n*=728, 42.3%) | Current/longstanding diagnosis | 295 (17.1%) |
| *Asian* |  |  | Previous diagnosis | 334 (19.4%) |
| North-East | 26 (1.5%) |  | Features without diagnosis | 99 (5.7%) |
| South-East | 25 (1.5%) | Bulimia Nervosa  (*n*=491, 28.5%) | Current/longstanding diagnosis | 151 (8.8%) |
| Southern/Central | 12 (0.7%) |  | Previous diagnosis | 252 (14.6%) |
| *European* |  |  | Features without diagnosis | 88 (5.1%) |
| Southern/Eastern | 430 (25.0%) | Binge Eating Disorder  (*n*=464, 26.9%) | Current/longstanding diagnosis | 171 (9.9%) |
| Northern/Western | 266 (15.4%) |  | Previous diagnosis | 143 (8.3%) |
| *Mixed/Diverse* | 28 (1.6%) |  | Features without diagnosis | 150 (8.7%) |
| ***Religious Affiliation*** |  | Other Specified Feeding and Eating Disorder/ Unspecified Feeding or Eating Disorder  (*n*=613, 35.6%) | Current/longstanding diagnosis | 238 (13.8%) |
| No religious affiliation | 729 (42.4%) |  | Previous diagnosis | 213 (12.4%) |
| Agnostic or atheist | 567 (32.9%) |  | Features without diagnosis | 162 (9.4%) |
| Christianity | 318 (18.4%) | Eating Disorder, unsure of specific diagnosis  (*n*=466, 27.0%) | Current/longstanding diagnosis | 238 (13.8%) |
| Paganism | 36 (2.1%) |  | Previous diagnosis | 95 (5.5%) |
| Spiritualism | 33 (1.9%) |  | Features without diagnosis | 133 (7.7%) |
| Judaism | 18 (1.0%) | ***Co-occurring mental health condition***^b^ | | *n (%)*^c^ |
| Buddhism | 12 (0.7%) | Anxiety Disorder | Current/longstanding diagnosis | 1,222 (70.9%) |
| Islam | 5 (0.3%) |  | Previous diagnosis | 190 (11.0%) |
| Hinduism | 2 (0.1%) | Depressive Disorder | Current/longstanding diagnosis | 951 (55.2%) |
| Prefer not to say | 3 (0.2%) |  | Previous diagnosis | 1225 (22.3%) |
| ***Location (State)*** |  | Obsessive Compulsive Disorder | Current/longstanding diagnosis | 1225 (71.1%) |
| Victoria | 786 (45.6%) |  | Previous diagnosis | 277 (16.1%) |
| New South Wales | 436 (25.3%) | Personality Disorder | | 236 (13.7%) |
| Queensland | 224 (13.0%) | Autism Spectrum Disorder | | 100 (5.8%) |
| Western Australia | 93 (5.4%) | History of alcohol or drug misuse | Currently | 226 (13.1%) |
| South Australia | 84 (4.9%) |  | Previously | 324 (18.8%) |
| Australian Capital Territory | 58 (3.4%) | Self-harm, suicidal ideation, or at least one suicide attempt | Currently | 426 (24.7%) |
| Tasmania | 37 (2.2%) |  | Previously | 698 (40.5%) |
| Northern Territory | 4 (0.2%) |  |  |  |
| ^a^Ethnicity based on Australian Standard Classification (Australian Bureau of Statistics, 2019). ^b^Multiple responses allowed  ^c^Percentage of total cohort (*N*=1723) | | | | |
