# Supplementary material for: The impact of the COVID-19 pandemic and associated public health response on people with eating disorder symptomatology: an Australian study
Source: J Eat Disord. 2022 Jan 17;10:9. doi: 10.1186/s40337-021-00527-0 (PMC8762631; doi:10.1186/s40337-021-00527-0)
Supplement: Supplementary file 4 — Additional file 4. Changes in eating disorder symptoms during the pandemic. [file 40337_2021_527_MOESM4_ESM.docx]

***The impact of the COVID-19 pandemic and associated public health response on people with eating disorder symptomatology: An Australian study***

| **Supplementary item 4:** Changes in eating disorder symptoms during the pandemic | | | | | | | |
| --- | --- | --- | --- | --- | --- | --- | --- |
| Eating Disorder symptom |  |  | Symptom change within pandemic | | | | |
|  | Pre-pandemic subgroup^a^ | *n* | Increased a lot | Increased somewhat | No change/NA | Decreased somewhat | Decreased a lot |
|  |  |  | *count (%)*^b^ | *count (%)*^b^ | *count (%)*^b^ | *count (%)*^b^ | *count (%)*^b^ |
| Body image concern | Current | 1565 | 1100 (70.3%) | 316 (20.2%) | 103 (6.6%) | 38 (2.4%) | 8 (0.5%) |
|  | Previous | 59 | 15 (25.4%) | 20 (33.9%) | 20 (33.9%) | 3 (5.1%) | 1 (1.7%) |
|  | I don’t know | 4 | 1 (25.0%) | 3 (75.0%) | 0 (0.0%) | 0 (0.0%) | 0 (0.0%) |
|  | Never/NA | 95 | 42 (44.2%) | 20 (21.1%) | 29 (30.5%) | 2 (2.1%) | 2 (2.1%) |
|  | Total | 1723 | 1158 (67.2%) | 359 (20.8%) | 152(8.8%) | 43 (2.5%) | 11 (0.6%) |
| Food restriction/dieting | Current | 1300 | 572 (44.0%) | 495 (38.1%) | 101 (7.8%) | 104 (8.0%) | 28 (2.2%) |
|  | Previous | 298 | 28 (9.4%) | 114 (38.3%) | 82 (27.5%) | 49 (16.4%) | 25 (8.4%) |
|  | I don’t know | 5 | 0 (0.0%) | 2 (40.0%) | 3 (60.0%) | 0 (0.0%) | 0 (0.0%) |
|  | Never/NA | 120 | 29 (24.2%) | 36 (30.0%) | 38 (31.7%) | 14 (11.7%) | 3 (2.5%) |
|  | Total | 1723 | 629 (36.5%) | 647 (37.6%) | 224 (13.0%) | 167 (9.7%) | 56 (3.3%) |
| Binge/over-eating | Current | 958 | 527 (55.0%) | 328 (34.2%) | 48 (5.0%) | 45 (4.7%) | 10 (1.0%) |
|  | Previous | 377 | 38 (10.1%) | 140 (37.1%) | 137 (36.3%) | 37 (9.8%) | 25 (6.6%) |
|  | I don’t know | 30 | 1 (3.3%) | 10 (33.3%) | 16 (53.3%) | 2 (6.7%) | 1 (3.3%) |
|  | Never/NA | 358 | 35 (9.8%) | 61 (17.0%) | 244 (68.2%) | 6 (1.7%) | 12 (3.4%) |
|  | Total | 1723 | 601 (34.9%) | 539 (31.3%) | 445 (25.8%) | 90 (5.2%) | 48 (2.8%) |
| Self-induced vomiting | Current | 430 | 190 (44.2%) | 124 (28.8%) | 75 (17.4%) | 24 (5.6%) | 17 (4.0%) |
|  | Previous | 484 | 16 (3.3%) | 74 (15.3%) | 359 (74.2%) | 12 (2.5%) | 23 (4.8%) |
|  | I don’t know | 14 | 0 (0.0%) | 2 (14.3%) | 12 (85.7%) | 0 (0.0%) | 0 (0.0%) |
|  | Never/NA | 795 | 14 (1.8%) | 17 (2.1%) | 761 (95.7%) | 0 (0.0%) | 3 (0.4%) |
|  | Total | 1723 | 220 (12.8%) | 217 (12.6%) | 1207 (70.1%) | 36 (2.1%) | 43 (2.5%) |
| Driven/over-exercise | Current | 639 | 308 (48.2%) | 194 (30.4%) | 59 (9.2%) | 56 (8.8%) | 22 (3.4%) |
|  | Previous | 597 | 43 (7.2%) | 199 (33.3%) | 196 (32.8%) | 92 (15.4%) | 67 (11.2%) |
|  | I don’t know | 51 | 1 (2.0%) | 11 (21.6%) | 31 (60.8%) | 4 (7.8%) | 4 (7.8%) |
|  | Never/NA | 436 | 18 (4.1) | 63 (14.4%) | 317 (72.7%) | 15 (3.4%) | 23 (5.3%) |
|  | Total | 1723 | 370 (21.5%) | 467 (27.1%) | 603 (35.0%) | 167 (9.7%) | 116 (6.7%) |
| Laxative and/or pill misuse | Current | 257 | 90 (35.0%) | 70 (27.2%) | 76 (29.6%) | 11 (4.3%) | 10 (3.9%) |
|  | Previous | 409 | 9 (2.2%) | 56 (13.7%) | 305 (74.6%) | 15 (3.7%) | 24 (5.9%) |
|  | I don’t know | 20 | 0 (0.0%) | 0 (0.0%) | 20 (100.0%) | 0 (0.0%) | 0 (0.0%) |
|  | Never/NA | 1037 | 8 (0.8%) | 19 (1.8%) | 999 (96.3%) | 0 (0.0%) | 11 (1.1%) |
|  | Total | 1723 | 107 (6.2%) | 145 (8.4%) | 1400 (81.3%) | 26 (1.5%) | 45 (2.6%) |
| Diet pill misuse | Current | 100 | 27 (27.0%) | 34 (34.0%) | 33 (33.0%) | 3 (3.0%) | 3 (3.0%) |
|  | Previous | 264 | 5 (1.9%) | 25 (9.5%) | 206 (78.0%) | 13 (4.9%) | 15 (5.7%) |
|  | I don’t know | 14 | 0 (0.0%) | 0 (0.0%) | 14 (100.0%) | 0 (0.0%) | 0 (0.0%) |
|  | Never/NA | 1345 | 2 (0.1%) | 8 (0.6%) | 1308 (97.2%) | 7 (0.5%) | 20 (1.5%) |
|  | Total | 1723 | 34 (2.0%) | 67 (3.9%) | 1561 (90.6%) | 23 (1.3%) | 38 (2.2%) |
| Other experiences |  | *n^c^* | Increased a lot | Increased somewhat | No change | Decreased somewhat | Decreased a lot |
|  |  |  | *count (%)^b^* | *count (%)^b^* | *count (%)^b^* | *count (%)^b^* | *count (%)^b^* |
| Quality of sleep |  | 1704 | 63 (3.7%) | 167 (9.8%) | 292 (17.1%) | 636 (37.3%) | 546 (32.0%) |
| Guilt buying food |  | 1654 | 980 (59.3%) | 466 (28.2%) | 175 (10.6%) | 22 (1.3%) | 11 (0.7%) |
| Alcohol use |  | 1368 | 305 (22.3%) | 383 (28.0%) | 479 (35.0%) | 99 (7.2%) | 102 (7.5%) |
| Smoking |  | 1018 | 181 (17.8%) | 147 (14.4%) | 590 (58.0%) | 39 (3.8%) | 61 (6.0%) |
| Recreational drug use |  | 1032 | 140 (13.6%) | 187 (18.1%) | 588 (57.0%) | 47 (4.6%) | 70 (6.8%) |
| Prescription medicine use |  | 1174 | 148 (12.6%) | 257 (21.9%) | 711 (60.6%) | 26 (2.2%) | 32 (2.7%) |
| *N*=total sample size; *n*=subsample size; NA=Not Applicable  ^a^Participants were asked to categorise the presence of each eating disorder symptom prior to the pandemic  ^b^Percentage of symptom subgroup  ^c^Denotes number of participants who reported this experience as applicable | | | | | | | |
